# Secondary hemophagocytic lymphohistiocytosis following bacterial pneumonia managed with dexamethasone monotherapy: a case report

**DOI:** 10.1097/MS9.0000000000005271

**Published:** 2026-06-19

**Authors:** Pukar KC, Aashish Kumar Jha, Nikita Gautam, Nishan Aryal, Bhushan Gautam, Lata Regmi, Devansh Joshi

**Affiliations:** a Department of Internal Medicine, Dhulikhel Hospital, Kathmandu University Hospital, Dhulikhel, Nepal; b Kathmandu University School of Medical Sciences, Dhulikhel, Nepal; c Department of Pathology, Dhulikhel Hospital, Kathmandu University Hospital, Dhulikhel, Nepal

**Keywords:** case report, cytokine storm, hemophagocytic lymphohistiocytosis, pneumonia, secondary HLH

## Abstract

**Introduction:**

Secondary hemophagocytic lymphohistiocytosis (HLH) is a rare hyperinflammatory syndrome in adults, resulting from uncontrolled immune activation and a cytokine storm, and is frequently misdiagnosed because its manifestations closely resemble severe infection or sepsis, particularly when triggered by bacterial pneumonia.

**Case presentation:**

A 63-year-old woman presented with 1 week of fever, cough, and dyspnea despite outpatient antibiotic treatment. Investigations confirmed right-sided lobar pneumonia caused by *Streptococcus pneumoniae*. Despite targeted intravenous antibiotics, she developed worsening cytopenias, hyperferritinemia, hypertriglyceridemia, and splenomegaly. Bone marrow aspiration revealed hemophagocytosis, confirming the diagnosis by fulfilling five out of the eight HLH-2004 criteria and an HScore of 200. Due to her age and treatment preferences, the patient was started on dexamethasone monotherapy. She showed rapid clinical and laboratory improvement within three days and remained stable during follow-up.

**Clinical discussion:**

Infection-associated HLH is commonly triggered by viral infections but can rarely occur secondary to bacterial infections, such as *S. pneumoniae*. HLH should be suspected when symptoms worsen despite appropriate antimicrobial therapy. Early recognition using the HLH-2004 criteria and HScore is vital for improving patient outcomes. Immunosuppressive therapy is essential to interrupt immune system activation and prevent organ failure. Although standard regimens include etoposide-based treatments, decisions must consider patient age, comorbidities, and tolerance.

**Conclusion:**

This case highlights the need for increased suspicion of HLH in patients with unremitting fever and progressive cytopenias, despite appropriate antimicrobial therapy. Dexamethasone monotherapy may be considered a potential alternative for select patients who cannot tolerate or do not consent to cytotoxic chemotherapy.

## Introduction

Hemophagocytic lymphohistiocytosis (HLH) is a severe hyperinflammatory syndrome characterized by uncontrolled immune activation, leading to excessive cytokine release and subsequent multiorgan dysfunction[1]. Early diagnosis and treatment are necessary to prevent mortality and morbidity, particularly neurological complications[2].

HLH can be classified into two types: primary (familial) and secondary HLH. Primary HLH is a genetic disorder that presents in infancy or early childhood, whereas secondary HLH occurs commonly in adults and is triggered by underlying conditions such as infections, malignancies, or autoimmune diseases^[^2–4^]^. Among these, infections are one of the most common precipitating factors^[^3,5–7^]^.

HIGHLIGHTS

- Secondary hemophagocytic lymphohistiocytosis (HLH) is a rare, life-threatening condition that follows malignancies, autoimmune conditions, and infections.
- Secondary HLH associated with *Streptococcus pneumoniae* infection is extremely uncommon and may be misdiagnosed as sepsis.
- Suspicion for HLH should be raised when persistent fever and progressive cytopenias are unresponsive to adequate antibiotic therapy.
- Timely recognition and appropriate immunosuppressive therapy can be life-saving.
- Dexamethasone monotherapy can be effective in cases where treatment with a standard regimen, including cytotoxic chemotherapy, is not feasible.

The pathophysiology of HLH involves impaired cytotoxic function of natural killer (NK) cells and CD8⁺ T lymphocytes, resulting in the failure to eliminate activated immune cells^[^1,8^]^. This leads to persistent immune stimulation and a cytokine storm, which manifests clinically as prolonged fever, cytopenias, hepatosplenomegaly, and markedly elevated inflammatory markers[3].

Diagnosing HLH in adults remains challenging due to its nonspecific presentation and significant overlap with severe infections and sepsis^[^9–11^]^. Infection-associated HLH, particularly when secondary to bacterial pneumonia, is rare and often remains undiagnosed[12]. We present a case of pneumonia-associated secondary HLH in an adult woman, emphasizing the need for early recognition in patients with persistent fever and cytopenias unresponsive to appropriate antimicrobial therapy. This case has been reported in line with the CARE guidelines[13].

## Case presentation

A 63-year-old woman with no significant medical or family history presented with 1 week of fever, cough, and dyspnea. She was empirically treated with azithromycin for 3 days without improvement before presentation. At presentation, she was febrile (temperature: 39°C), with tachycardia (pulse: 134/min) and tachypnea (respiratory rate: 25/min). The patient had crackles on auscultation. Initial laboratory investigations demonstrated leukocytosis with bicytopenia (anemia and thrombocytopenia), elevated inflammatory markers, and radiographic evidence of lobar consolidation involving the right lung field. Gram stain of the sputum revealed gram-positive cocci in pairs, and sputum culture was positive for *Streptococcus pneumoniae*, which confirmed the diagnosis of bacterial pneumonia.

The patient was initially treated with intravenous piperacillin and tazobactam, which was changed to meropenem after the sputum culture and antibiotic sensitivity report. However, the patient did not show clinical improvement; hence, a CT scan was performed, which revealed lobar consolidation, pleural effusion, and splenomegaly. Repeat blood analysis showed high ferritin, decreasing hemoglobin and platelet counts, and a persistently elevated CRP.

**Figure 1.**
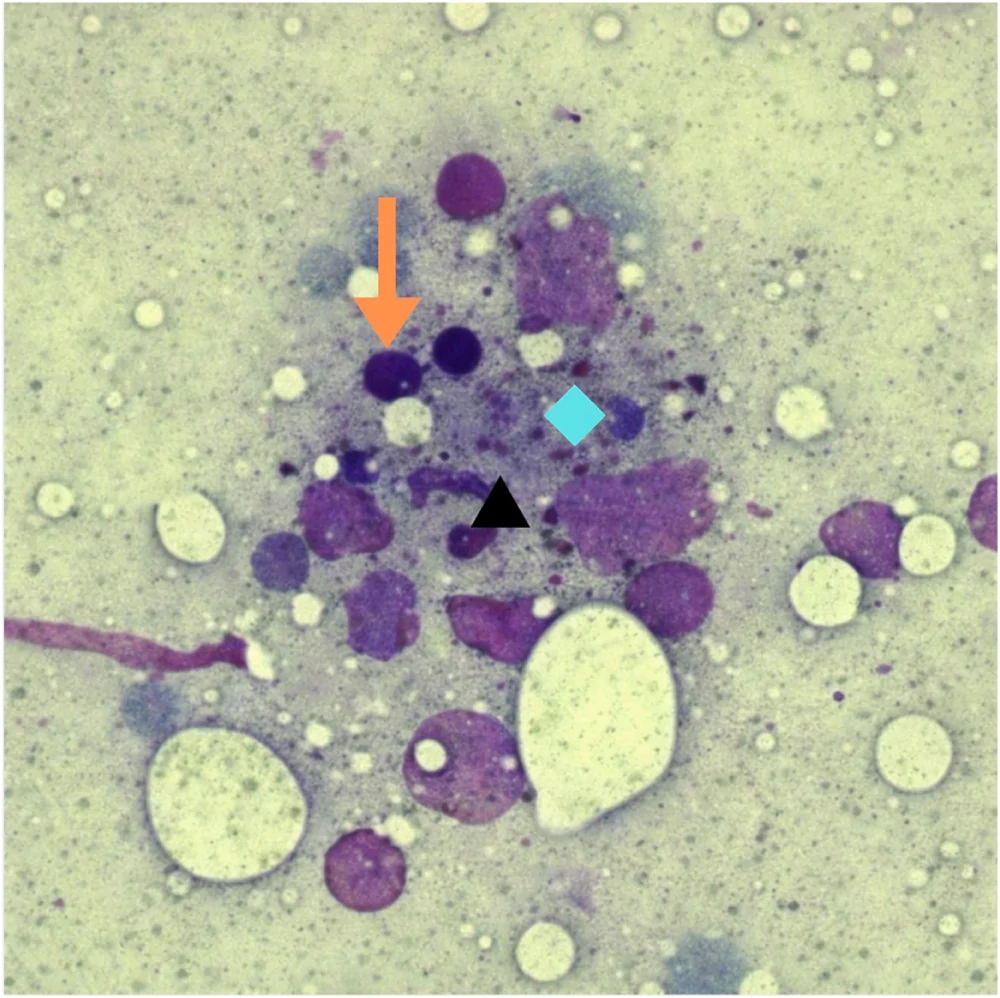
Bone marrow aspirate smear demonstrating hemophagocytosis. The smear shows a reactive histiocyte with phagocytosed neutrophils (indicated by ▲), erythroid precursors (indicated by ↓), and platelets (indicated by ◆).

**Figure 2.**
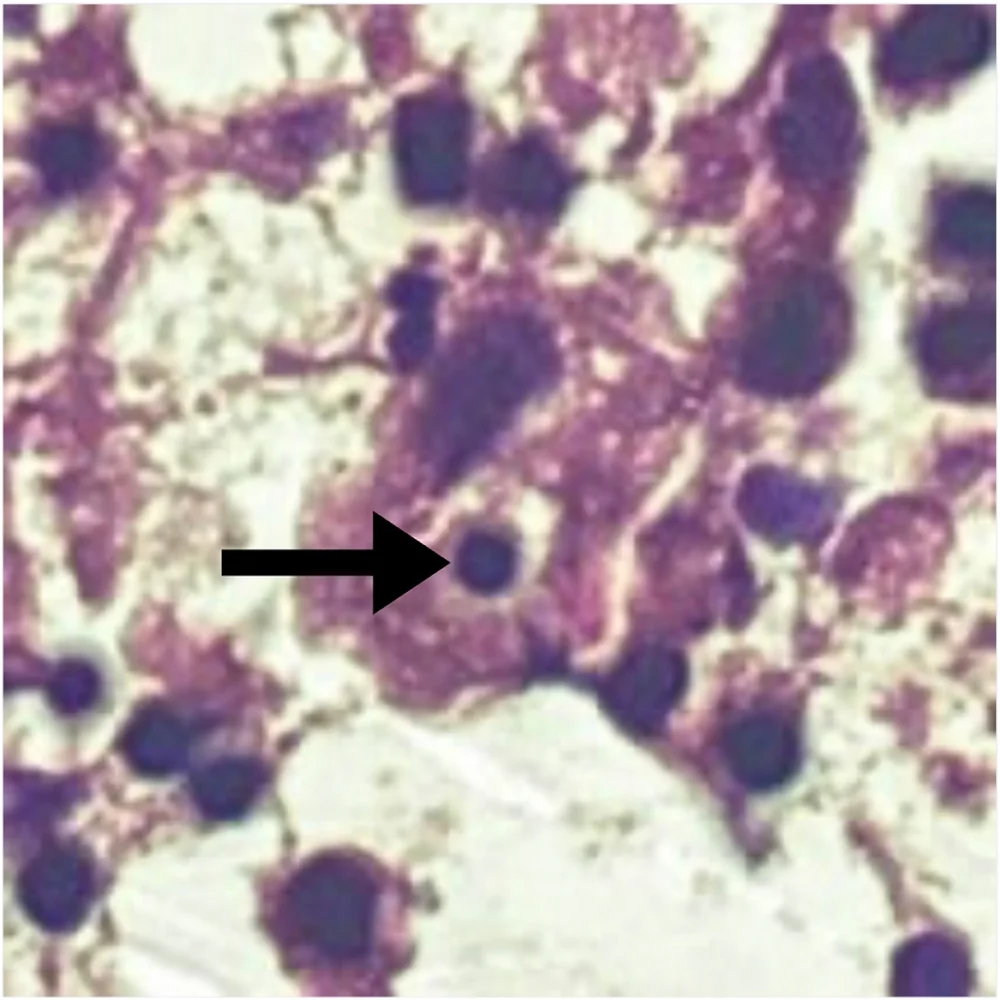
Bone marrow biopsy section showing evidence of hemophagocytic activity. The biopsy demonstrates a histiocyte containing a phagocytosed erythroid precursor (indicated by →).

The combination of high ferritin, bicytopenia, splenomegaly, persistent fever, and raised CRP, as mentioned in Table 1, led to the suspicion of secondary HLH. Bone marrow aspiration (Fig. 1) and biopsy (Fig. 2) showed reactive histiocytes phagocytosing hematopoietic elements consisting of erythroid precursors, platelets, and neutrophils. As five out of the eight HLH-2004 diagnostic criteria were fulfilled, and an H-Score of 200 points was calculated–which corresponds to an 88% probability of HLH[14], the diagnosis of HLH was established. The patient refused the use of etoposide after being informed about its possible adverse effects. Drawing on the elements of the HLH-94 guidelines, we initiated a once-daily intravenous dose of 20 mg dexamethasone (∼10 mg/m^2^). After administering this full dose for 2 weeks, a tapering phase was introduced, allowing the medication to be completely discontinued by week eight.

**Table 1:** Timeline of clinical course, laboratory findings, and management.

Timepoint	Clinical status and interventions	Laboratory and imaging parameters
~1 month prior	Symptom onset: Patient developed fevera, productive cough, and dyspnea.	N/A
Day 1	Initial presentation: Evaluated at a rural primary care center.	Hb: 11.2 g/dL (↓)
		Platelets: 108 × 10^3^/μL (↓)
	Started on empirical oral azithromycin and IV ceftriaxone.	
		Neutrophils: 4.0 × 10^3^/μL (64.5%)
Day 3	Referral to a tertiary care center due to lack of improvement.	Hb: 9.5 g/dL (↓)
		Platelets: 150 × 10^3^/μL (↑)
	Escalated to IV piperacillin-tazobactam.	
	Microbiology: Sputum Gram stain showed gram-positive cocci in pairs.	Neutrophils: 3.25 × 10^3^/μL (65%) (↓)
Days 4–6	Suspicion of HLH: Clinical suspicion due to marked hyperferritinemia and splenomegaly.	Ferritin: >2000 ng/mL† (↑↑)
		Triglycerides: 276 mg/dL† (↑)
		CRP: >90 mg/L (↑↑)
		Imaging (USG): Splenomegaly (15 cm)†
Days 7–9	Clinical worsening: Escalated to IV meropenem due to unremitting fevera and suspected complicated pneumonia.	Worsening bicytopenia†
		Hb: 8.8 g/dL (↓)
		Platelets: 96 × 10^3^/μL (↓)
		Neutrophils: 2.34 × 10^3^/μL (67%) (↓)
Day 13	Diagnosis and intervention: Bone marrow biopsy confirmed prominent hemophagocytosisa and secondary HLH diagnosis.	Hb: 6.9 g/dL (↓)
		Platelets: 89 × 10^3^/μL (↓)
		Neutrophils: 1.65 × 10^3^/μL (59%) (↓)
	Commenced IV dexamethasone (20 mg daily).	Triglycerides: 636 mg/dL† (↑↑)
		Ferritin: >2000 ng/mL† (↑↑)
		CRP: >90 mg/L (↑↑)
Day 16	Follow-up and discharge: Clinical and laboratory improvement.	Hb: 7.5 g/dL (↑)
	Discharged on a tapering dose of oral dexamethasone.	Platelets: 135 × 10^3^/μL (↑)
		Neutrophils: 4.4 × 10^3^/μL (80%) (↑)
		Ferritin: >1000 ng/mL (↓)
		CRP: 41.0 mg/L (↓)

N/A, not available; IV, intravenous; Hb, hemoglobin; HLH, hemophagocytic lymphohistiocytosis; CRP, C-reactive protein; USG, ultrasonography.

†A confirmed HLH-2004 diagnostic criterion was met.

Arrows denote laboratory value trends: (↑) elevated/increasing; (↓) decreased/decreasing; (↑↑) markedly elevated.

The patient demonstrated rapid clinical improvement following dexamethasone therapy, including a decrease in CRP, remission of fever, and partial recovery of hemoglobin and platelet counts within 3 days (Table 1). She was discharged in 5 days with oral medications and a regular follow-up plan. During her follow-up visits at weeks 2, 6, and 8, she remained afebrile, and no recurrence of respiratory symptoms was observed. The cytopenias improved, with resolution of thrombocytopenia, hypertriglyceridemia, and high ferritin levels.

## Discussion

HLH secondary to infection is most commonly associated with Epstein–Barr Virus. It is uncommon in bacterial infections, but when present, it is associated with tuberculosis. Very few cases of HLH secondary to *S. pneumoniae* have been reported to date^[^1,2,15–17^]^. Our case adds to the growing literature by describing HLH secondary to pneumonia caused by *S. pneumoniae*.

The epidemiological burden of secondary HLH has not been established accurately, as most cases remain largely underdiagnosed worldwide[2]. However, the combined incidence of primary and secondary HLH in England was reported to be 4.2 cases per million population in 2018[18].

The diagnosis of HLH remains a major clinical challenge, as its presentation frequently overlaps with conditions that must be considered both as differential diagnoses and potential triggers for HLH[19]. Although the revised HLH-2024 guidelines have been published for familial HLH, the diagnosis of secondary HLH is complicated by the lack of specific and definitive diagnostic tests[20]. The HScore proposed by Fardet *et al*[14] and the HLH-2004 criteria serve as frameworks for the diagnosis and management of secondary HLH[21].

As per the HLH-2004 guidelines, the diagnosis of HLH can be established if at least 5 of the 8 diagnostic criteria are fulfilled. Diagnostic criteria include fever (peak temperature of >38.5°C for >7 days), splenomegaly, cytopenias involving >2 cell lines (hemoglobin <9 g/dL, platelets <100,000/μL, neutrophils <1000/μL), hypertriglyceridemia (fasting triglycerides >265 mg/dL) or hypofibrinogenemia (fibrinogen <1.5 g/L), elevated soluble interleukin-2 receptor (sCD25) levels (>2400 U/mL)[21]. Likewise, the HScore is a scoring system to determine the probability of HLH based on nine parameters, including known underlying immune suppression, temperature (°C), organomegaly, number of cytopenias, levels of ferritin (μg/L), triglycerides (mmol/L), fibrinogen (g/L), aspartate transaminase (U/L), and hemophagocytosis features on bone marrow aspirate[14].

The early initiation of treatment for HLH with immunochemotherapy, including etoposide, dexamethasone, and cyclosporine A, has significantly improved the prognosis^[^20,22^]^. Guidelines for the treatment of secondary adult HLH recommend individualized treatments that incorporate elements of the HLH-94 guidelines[9]. However, the use of chemotherapeutic agents, including etoposide, contributes to unwanted toxicity, especially in the elderly, making them vulnerable to end-organ damage[9]. Intravenous immunoglobulin and corticosteroid therapy have been studied as an effective alternative for HLH treatment with minimal toxicity[23].

Dexamethasone exerts its critical immunosuppressive effects by diffusing across cell membranes and binding to the glucocorticoid receptor, which subsequently downregulates the NF-*κ*B signaling pathway. This further inhibits the transcription and release of key pro-inflammatory cytokines, including IFN-γ, IL-6, and TNF-α, which are central to the pathogenesis of HLH[24].

In this case, dexamethasone therapy resulted in rapid improvement of the clinical condition, as well as laboratory parameters, and provided symptomatic relief for the patient. Taking into account her age, the patient did not consent to the use of etoposide, considering her vulnerability to end-organ damage and other risks of HLH chemotherapy[9].

The patient’s initial presentation, with laboratory and radiological evidence, directed the treatment toward complicated pneumonia with antibiotics. However, consistent cytopenias, hyperferritinemia, and hypertriglyceridemia, despite appropriate and escalated antibiotic therapy, shifted suspicion from severe sepsis to hyperinflammatory conditions. Macrophage activation syndrome and malignancy-associated HLH were considered as key differentials but were less likely due to the absence of rheumatologic history and a bone marrow aspirate lacking malignant cells, respectively. This demonstrates that diagnosing HLH can be challenging due to its nonspecific and overlapping symptoms, yet early diagnosis is crucial for timely management. Early identification and management of HLH with immunochemotherapy improve the prognosis of the patient.

We acknowledge several limitations in this report. The diagnostic workup was limited by the unavailability of specialized laboratory tests such as sCD25 and NK cell activity testing, and the lack of reported fibrinogen levels for the assessment of hypofibrinogenemia. Additionally, we cannot entirely exclude the possibility of spontaneous clinical improvement and the long-term future risk of HLH relapse. As a single case study, our findings are hypothesis-generating and cannot be generalized to a broader patient population.

## Conclusion

Clinicians must maintain a high index of suspicion for secondary HLH in bacterial infections, such as streptococcal pneumonia, when patients present with unremitting fever and progressive cytopenias despite adequate antibiotic therapy. While the etoposide and dexamethasone combination regimens are standard, this case highlights how dexamethasone monotherapy may serve as an alternative therapeutic option in patients who cannot tolerate or do not consent to receive cytotoxic chemotherapy. Early recognition using the HScore and HLH-2004 criteria can improve prognosis and reduce mortality.

## Data Availability

All relevant data are included within the article. The data supporting the findings of this study are available from the corresponding author upon reasonable request.
